# Association of glucagon-like peptide-1 (GLP-1) receptor agonists and diabetic retinopathy (DR) – a systematic review and meta-analysis

**DOI:** 10.3389/fmed.2025.1639704

**Published:** 2025-12-18

**Authors:** Hassan Alwafi, Sarah Saleh Al-Harbi, Ghada Ahmad Aladwani, Safaa M. Alsanosi, Tope Oyelade, Fahd Almalki, Husna Irfan Thalib, Abdallah Y. Naser, Manal Z. Alfahmi, Basil AlOtaibi, Samra Fuad, Mohammed Talha Mohammed Zubair, Abdulelah M. Aldhahir, Widya N. Insani, Abdullah A. Alqarni, Jaber S. Alqahtani, Deema S. Ashoor, Mohammad Saleh Dairi

**Affiliations:** 1 Department of Pharmacology and Toxicology, College of Medicine, Umm Al-Qura University, Makkah, Saudi Arabia; 2 Department of Pharmacy, Umm Al-Qura University, Makkah, Saudi Arabia; 3 Institute for Liver and Digestive Health, UCL Division of Medicine, University College London, London, United Kingdom; 4 Department of Medicine, College of Medicine, Umm Al-Qura University, Makkah, Saudi Arabia; 5 General Medicine Practice Program, Batterjee Medical College, Jeddah, Saudi Arabia; 6 Department of Applied Pharmaceutical Sciences and Clinical Pharmacy, Faculty of Pharmacy, Isra University, Amman, Jordan; 7 Executive Administration of Research and Innovation, King Abdallah Medical City, Makkah, Saudi Arabia; 8 Independent Researcher, Jeddah, Saudi Arabia; 9 Respiratory Therapy Program, Department of Nursing, College of Nursing and Health Sciences, Jazan University, Jazan, Saudi Arabia; 10 Department of Pharmacology and Clinical Pharmacy, Padjadjaran University, Jatinangor, Indonesia; 11 Department of Respiratory Therapy, Faculty of Medical Rehabilitation Sciences, King Abdulaziz University, Jeddah, Saudi Arabia; 12 Department of Respiratory Care, Prince Sultan Military College of Health Sciences, Dammam, Saudi Arabia; 13 Al-Noor Specialist Hospital, Ministry of Health, Makkah, Saudi Arabia

**Keywords:** GLP-1 agonist, diabetes, obesity, diabetic retinopathy, meta-analysis, systematic review

## Abstract

**Objectives:**

Previous studies have shown conflicting results on the relationship between glucagon-like peptide-1 (GLP-1) receptor agonists and diabetic retinopathy (DR). This systematic review and meta-analysis aimed to clarify the association between GLP-1 receptor agonists use and the development or progression of DR.

**Methods:**

A comprehensive search of MEDLINE (via OVID and PubMed), Embase, Cochrane Central Register of Controlled Trials, and ClinicalTrials.gov was conducted from inception to March 2025. We included randomized controlled trials (RCTs) and observational studies reporting on the association between GLP-1 receptor agonists and DR. Screening, data extraction, and quality appraisal were performed independently and in duplicate. We assessed study quality using the Cochrane risk-of-bias tool for RCTs and the Newcastle-Ottawa Scale for observational studies. Meta-analysis was conducted using Stata 17, following PRISMA and MOOSE guidelines.

**Results:**

The search identified 6,922 studies. Of these, 39 articles (24 RCTs and 15 observational studies) met the inclusion criteria and 23 were included in the meta-analysis. The pooled analysis showed that GLP-1 receptor agonists were not significantly associated with the risk of DR compared with comparators (pooled RR = 1.00, 95% CI 0.71–1.43). Subgroup analyses by study design yielded similar non-significant results, with a pooled RR of 0.91 (95% CI 0.73–1.14) for randomized controlled trials and 2.09 (95% CI 0.47–9.19) for observational studies. After excluding studies with a high risk of bias, the pooled estimate remained non-significant (RR = 1.06, 95% CI 0.67–1.67), supporting the robustness of the overall findings. The association remained non-significant when restricted to larger studies (>500 participants; RR = 1.13, 95% CI 0.70–1.84).

**Conclusion:**

In conclusions, this systematic review found no significant association between GLP-1 receptor agonists and DR risk, though a non-significant trend toward lower risk was observed in randomized trials. Given the limited number of long-term studies, the current evidence remains inconclusive. Future studies with longer follow-up period are warranted to clarify the long-term ocular safety of GLP-1 receptor agonists.

**Systematic review registration:**

https://www.crd.york.ac.uk/PROSPERO/view/CRD420251007882.

## Highlights

- No significant association was found between GLP-1 receptor agonist use and the overall risk of developing or progressing diabetic retinopathy (pooled RR = 1.00, 95% CI 0.71–1.43). Sensitivity analysis excluding studies with high-risk of bias yielded a similar non-significant estimate (RR = 1.06, 95% CI 0.67–1.67), suggesting that the findings are robust.
- Subgroup analyses by study design also showed non-significant results, with a trend toward lower risk in randomized controlled trials (RR = 0.91, 95% CI 0.73–1.14) and higher but non-significant risk in observational studies (RR = 2.09, 95% CI 0.47–9.19). The association remained non-significant even when restricted to larger studies (>500 participants; RR = 1.13, 95% CI 0.70–1.84).
- While current evidence shows no significant risk, long-term safety data beyond 1–2 years remain limited. Clinicians should closely monitor patients with pre-existing retinopathy and ensure gradual glycemic improvement with coordinated ophthalmologic care until long-term safety is confirmed.

## Introduction

Diabetes mellitus (DM) is one of the most common diseases worldwide. It is a metabolic disorder characterized by high blood glucose levels resulting from various pathogenic processes, including insufficient insulin secretion, resistance to insulin action, or both (1, 2).

The two primary classifications of DM are type 1 diabetes mellitus (T1DM) and type 2 diabetes mellitus (T2DM) (1, 2).

The management of diabetes and the development of antidiabetic drugs have significantly evolved in recent years. The primary goal of diabetes therapy is to achieve optimal glycemic control while preventing both acute and chronic complications (3). One such chronic complication is diabetic retinopathy (DR), a major microvascular disorder caused by prolonged hyperglycemia, leading to visual impairment and blindness. DR develops due to structural and functional changes in the retinal vasculature and blood flow properties (4). Approximately one-third of individuals with diabetes develop DR, and among them, about one-third may progress to proliferative DR (5, 6). The incidence of DR is projected to increase from 415 million in 2015 to 642 million by 2040 (7). Regular screening, including annual dilated eye exams starting at diagnosis in T2DM and after 5 years in T1DM, is recommended to detect DR early and prevent progression (8).

Glucagon-like peptide-1 (GLP-1) receptor agonists have been available since 2005, with exenatide being the first medication in this class (9). These agents effectively lower blood glucose levels by enhancing glucose-dependent insulin secretion, suppressing glucagon release, and slowing gastric emptying, ultimately improving glycemic control with minimal risk of hypoglycemia (10, 11). Notably, GLP-1 receptors are highly expressed in the human retina (12).

This has led to the hypothesis that GLP-1 receptor agonists may have a protective role in preventing DR. However, the available evidence remains limited and contradictory (13–16).

Recent studies have shown that GLP-1 receptor agonists can improve the prognosis of DR (17, 18). However, the REWIND (19) and LEADER (20) trials found no significant association between GLP-1 receptor agonist use and DR. In contrast, the SUSTAIN-6 trial reported a higher incidence of DR in the GLP-1 receptor agonist group compared to the placebo group (13).

This systematic review aims to investigate the relationship between DR outcomes and GLP-1 receptor agonist treatment. It is hypothesized that the use of GLP-1 receptor agonists may reduce the risk of DR in patients with DM compared to placebo or other antidiabetic agents.

## Methods

The protocol for this systematic review and meta-analysis was registered in the International Prospective Register of Systematic Reviews (PROSPERO) (CRD42023493781). All findings were reported in accordance with the Preferred Reporting Items for Systematic Reviews and Meta-Analyses (PRISMA) guidelines statement.

### Search strategy

We searched Medline, Embase, ClinicalTrials.gov, and Cochrane from inception to March 2025 using a pre-defined search strategy. The search was conducted by a professional research librarian using both controlled vocabulary and sensitive keyword terms: “Diabetic,” “Obesity,” “liraglutide,” “exenatide,” “albiglutide,” “taspoglutide,” “dulaglutide,” “lixisenatide,” “semaglutide,” “efpeglenatide,” “cotadutide,” “GLP-1,” “toxic,” “overdose,” “safety,” and “diabetic retinopathy.” Related subject headings for each database were also included. Only English-language articles were included, and studies were excluded if they were animal studies, conference abstracts without full text, or did not report safety outcomes. A PRISMA checklist is provided as Supplementary Table S1.

### Study selection

We sought full-text published manuscripts of RCTs and observational studies using a predefined Population/Intervention/Comparator/Outcome (PICO) framework. Our population of interest included adult patients (≥18 years) with diabetes. The intervention was the use of GLP-1 receptor agonists (such as liraglutide, exenatide, semaglutide, etc.). Our comparator was either other anti-diabetic agents or placebo. The primary outcome was the development or progression of DR. Secondary outcomes included adverse effects associated with GLP-1 receptor agonists, such as toxicity, overdose, and any retinopathy-related outcomes. We excluded studies involving case series, case reports, review articles, pediatric populations, and those not involving GLP-1 receptor agonists.

### Study screening

**Figure 1:**
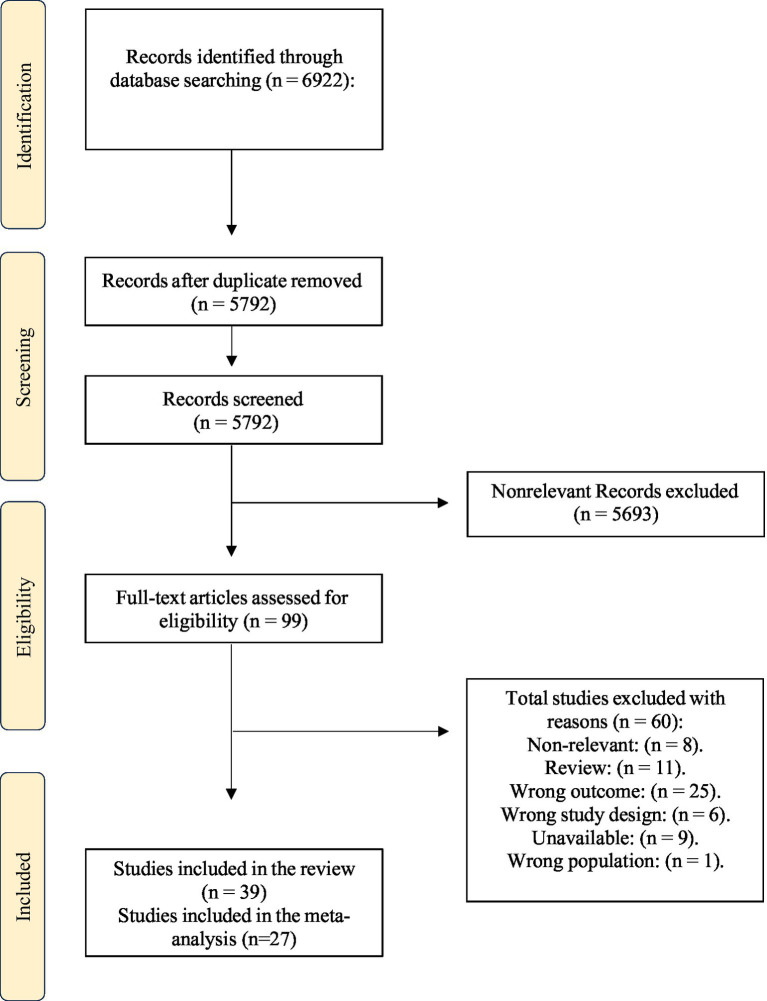
Flow diagram describing selection of trials for meta-analysis.

All articles were screened by two reviewers (A.B and H.A) independently and in parallel using titles and abstracts. If pre-defined eligibility criteria were met, articles were retrieved in full by two reviewers (S.A and G.A). Any discrepancies at the screening phase were resolved under the supervision of a third reviewer (R.K) through discussion, and consensus (Figure 1).

Cross-references of all retrieved manuscripts were also checked to identify additional studies.

### Data extraction

The data collection form was designed after taking into consideration how much information should be collected. All data from each eligible study was entered into a standardized spreadsheet in Excel 2010, this includes characteristics and outcomes of the studies included (Supplementary Table S2), and quality assessment of the studies included (Table 1). All the information in this study was collected and extracted by two authors (S.A and G.A) and was then cross-checked. Specifically, the general information in every study includes the following: title, author, year of publication, study design, study duration, sample size, gender, ethnicity, mean age, GLP-1-based therapy, control medication, study site, outcome of the study, and quality assessment. The outcome of the study in general includes the summary of the outcome information including the association of the use of GLP-1 agonist and risk of DR.

**Table 1:** Quality assessment for cohort studies.

Newcastle-Ottawa Scale (NOS) – for cohort study/Item and score										Categories
Study ID	Representiveness of the exposed cohort (1)	Selection of the non-exposed cohort (1)	Ascertainment of exposure (1)	Demonstration that outcome of interest was not present at start of study (1)	Compare ability of cohorts on the basis of the design or analysis (2)	Assessment of outcome (1)	Was follow up long enough for outcomes to occur (1)	Adequacy of follow up of cohorts (1)	Total	
Thomas Arendt Nielsen, 2022 (21)	–	*	*	–	**	*	–	–	5	Fair quality
Jennieh Best, 2011 (23)	*	*	*	–	**	*	*	*	8	Good quality
M. Angelyn Bethel, 2020 (24)	*	*	*	-	**	-	*	*	7	Good quality
Blaslov, K. 2013 (25)	–	*	*	*	**	*	–	*	7	Good quality
Daniel G. Dauner 2021 (27)	*	–	*	*	–	*	–	–	4	Fair quality
Jaime A. Davidson 2017 (28)	–	*	*	*	**	*	*	–	7	Good quality
Antonios Douros, 2018 (17)	*	*	*	*	**	*	*	*	9	Good quality
Lixin Guo. 2022 (34)	*	*	*	–	**	*	–	*	7	Good quality
Yuan Lin, 2023 (35)	*	*	*	–	**	*	*	*	8	Good quality
Gian Paolo Fadini, 2018 (41)	*	*	*	–	**	-	*	–	6	Fair quality
Tzu-Yi Lin, 2023 (43)	–	*	*	*	**	*	*	*	8	Good quality
Ueda, P. 2019 (15)	*	*	*	–	**	*	*	*	8	Good quality
Wang, T. 2018 (44)	–	*	*	–	**	*	*	*	7	Good quality
Zheng, D. 2023 (45)	*	*	*	*	**	*	*	*	9	Good quality
Sean D Sullivan, 2009 (49)	*	*	*	–	**	–	*	*	7	Good quality

Good quality: 3 or 4 stars in selection domain AND 1 or 2 stars in comparability domain AND 2 or 3 stars in outcome/exposure domain. Fair quality: 2 stars in selection domain AND 1 or 2 stars in comparability domain AND 2 or 3 stars in outcome/exposure domain. Poor quality: 0 or 1 star in selection domain OR 0 stars in comparability domain OR 0 or 1 stars in outcome/exposure domain.

### Quality assessment

Two reviewers independently assessed the risk of bias for each included study (S.A. and G.A.). Any disagreements were resolved by a third author (R.K.). The risk of bias was assessed for observational studies using the Newcastle-Ottawa Scale of Quality Assessment, which includes six criteria: representativeness of the exposure, selection of non-exposed cohorts, ascertainment of exposure, absence of the outcome of interest at the start of the study, comparability of cohorts based on design or analysis, assessment of the outcome, follow-up length, and loss to follow-up rate. For randomized clinical trials (RCTs), we used the Cochrane Collaboration’s Risk of Bias tool, which evaluates domains such as random sequence generation, allocation concealment, blinding, incomplete outcome data, selective reporting, and other biases.

### Statistical analysis

Dichotomous outcomes were extracted as events/non-events, while continuous outcomes were extracted as means and standard deviations. Effect estimates were summarized using forest plots, and statistical heterogeneity was assessed using the *I*^2^ statistic. A random-effects model was applied to account for between-study variability. Subgroup analyses were conducted based on study design (randomized controlled trials and observational studies), comparator type (insulin and other antidiabetic drugs) and GLP-1 types. Two sensitivity analyses were also conducted by excluding studies with high risk of bias and those with a total sample size of <500 participants to evaluate the robustness of the pooled estimates. Publication bias was assessed visually using funnel plots and statistically using Egger’s regression test. Data analysis was performed using Stata 17 software.

## Results

### Study characteristics

The initial literature search identified 6,922 publications. After excluding duplicates, 5,792 records remained. In total, 99 articles were identified as potentially eligible based on abstract review. After a full-text review of the 99 eligible studies, 55 studies were excluded. In the end, 39 studies were included for the systematic review and 27 articles were considered appropriate for inclusion in the meta-analysis, as shown in Figure 1. Characteristics of all the 39 included studies are displayed in Supplementary Table S2 (13, 15, 17, 21–56).

### Outcomes

#### Association of GLP-1 receptor agonists and DR

**Figure 2:**
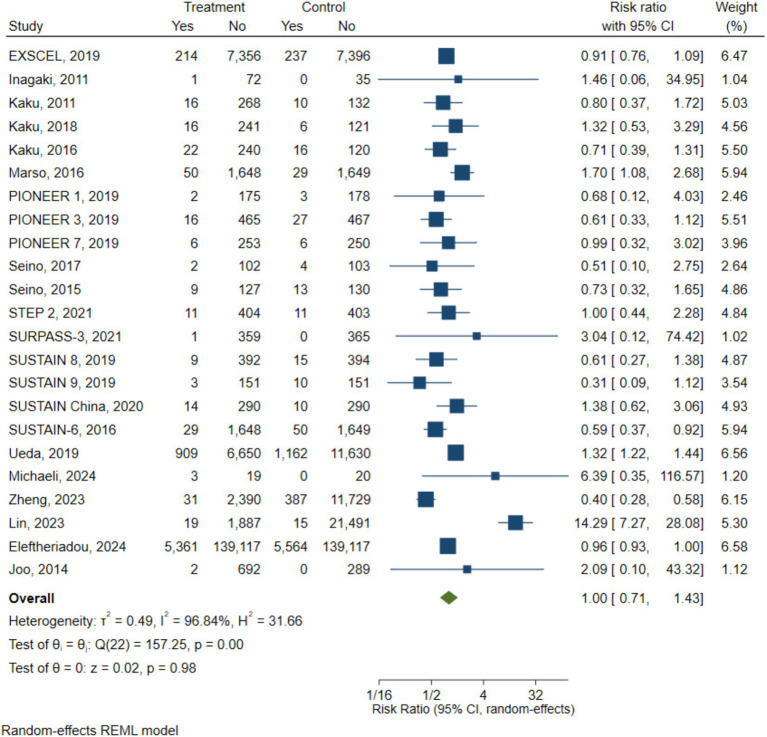
Pooled risk ratios (95% CI) for diabetic retinopathy comparing GLP-1 receptor agonists with control groups under a random-effects model.

Our findings have shown that no significant association was found between GLP-1 receptor agonist use and the overall risk of developing or progressing DR (pooled RR = 1.00, 95% CI 0.71–1.43) (Figure 2). However, substantial heterogeneity was observed across studies (*I*^2^ = 96.8%), indicating considerable variability in study results. To explore the potential sources of heterogeneity, subgroup analyses were conducted based on study design, comparator type, and GLP-1 types.

##### Subgroup analysis

**Figure 3:**
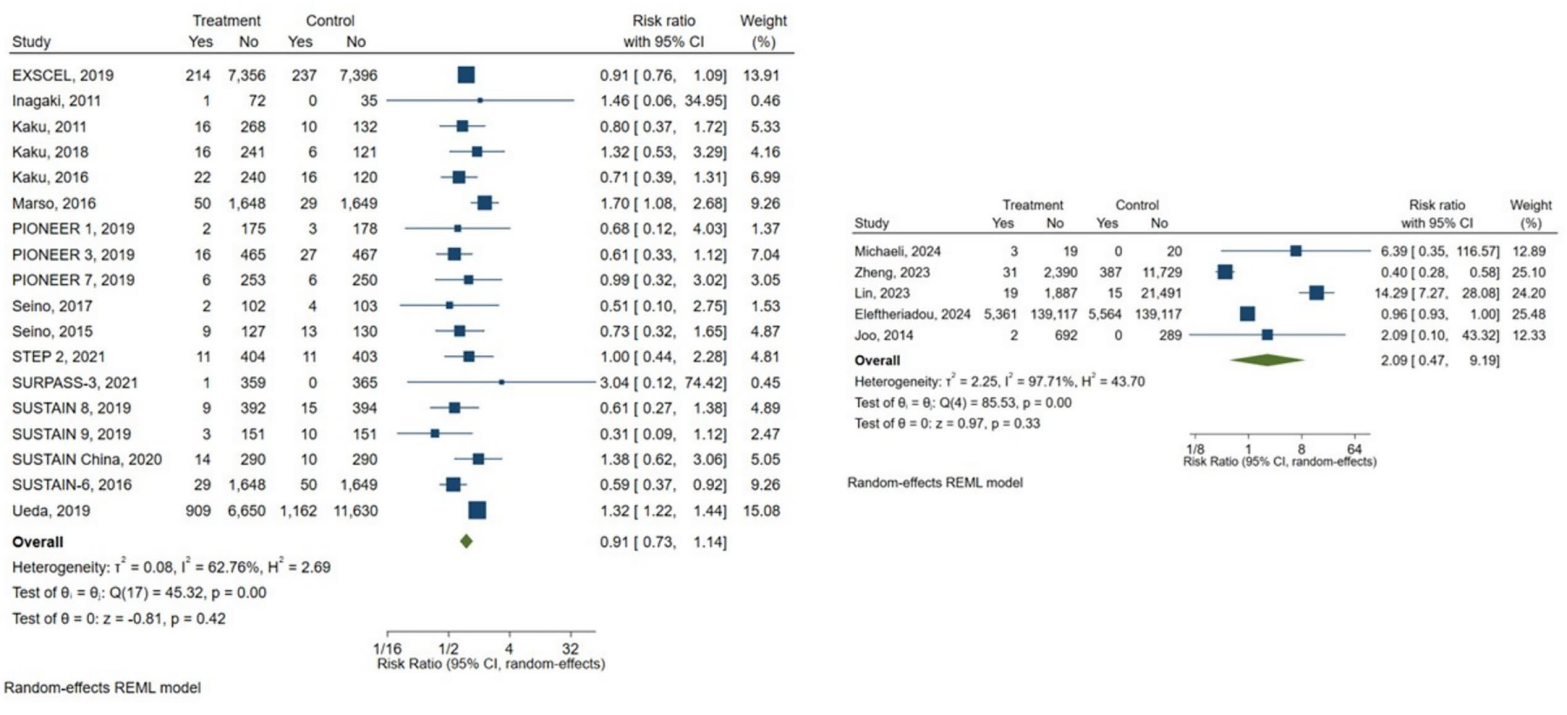
Subgroup analysis comparing randomized controlled trials and observational studies.

Subgroup analyses by study design also demonstrated non-significant findings across both randomized controlled trials and observational studies. Among randomized controlled trials, the pooled risk ratio was (RR = 0.91, 95% CI 0.73–1.14), suggesting a non-significant trend toward lower risk compared with control treatments, with low-to-moderate heterogeneity (*I*^2^ = 62.76%). In contrast, observational studies yielded a higher but imprecise pooled estimate (RR = 12.09, 95% CI 0.47–9.19), reflecting wider variability and possible residual confounding (Figure 3).

**Figure 4:**
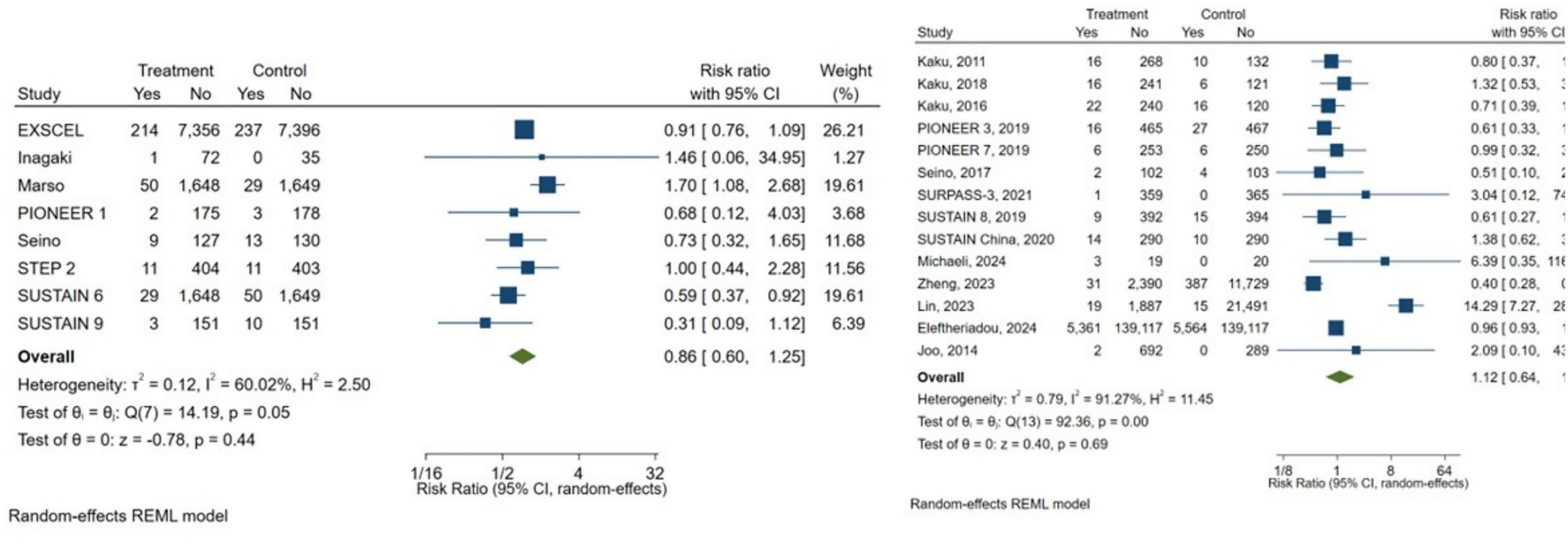
Subgroup analysis comparing comparator groups (placebo vs. other antidiabetic drugs).

Given the variability in comparator types across studies, subgroup analyses were performed according to the comparator group. Studies comparing GLP-1 receptor agonists with placebo showed protective but no significant association with DR risk (pooled RR = 0.86, 95% CI 0.60–1.25; *I*^2^ = 60.02%). Similarly, studies using other antidiabetic agents as comparators (including SGLT2 inhibitors, DPP-4 inhibitors, and sulfonylureas) also showed a non-significant association (pooled RR = 1.12, 95% CI 0.64–1.96; *I*^2^ = 91.27%) (Figure 4).

Semaglutide was the most frequently investigated GLP-1 receptor agonist among the included studies. In a subgroup analysis restricted to semaglutide, the pooled estimate showed no significant association with DR risk compared with placebo or other antidiabetic drugs (RR = 0.86, 95% CI 0.62–1.20; *I*^2^ = 45.5%), suggesting moderate heterogeneity and a trend toward a protective but non-significant effect. An additional subgroup analysis was performed to explore the ocular safety profile of semaglutide compared with other GLP-1 receptor agonists. The pooled estimate showed no significant difference in the risk of DR (RR = 0.79, 95% CI 0.34–1.80; *I*^2^ = 0%), indicating consistency across studies and suggesting that semaglutide has a comparable safety profile to other agents in this class (Supplementary Figures S1, S2).

##### Sensitivity analysis

Sensitivity analysis excluding studies with high-risk of bias yielded a similar non-significant estimate (RR = 1.06, 95% CI 0.67–1.67), suggesting that the findings are robust. The association remained non-significant even when restricted to larger studies (>500 participants; RR = 1.13, 95% CI 0.70–1.84) (Supplementary Figures S3, S4).

##### Publication bias

**Figure 5:**
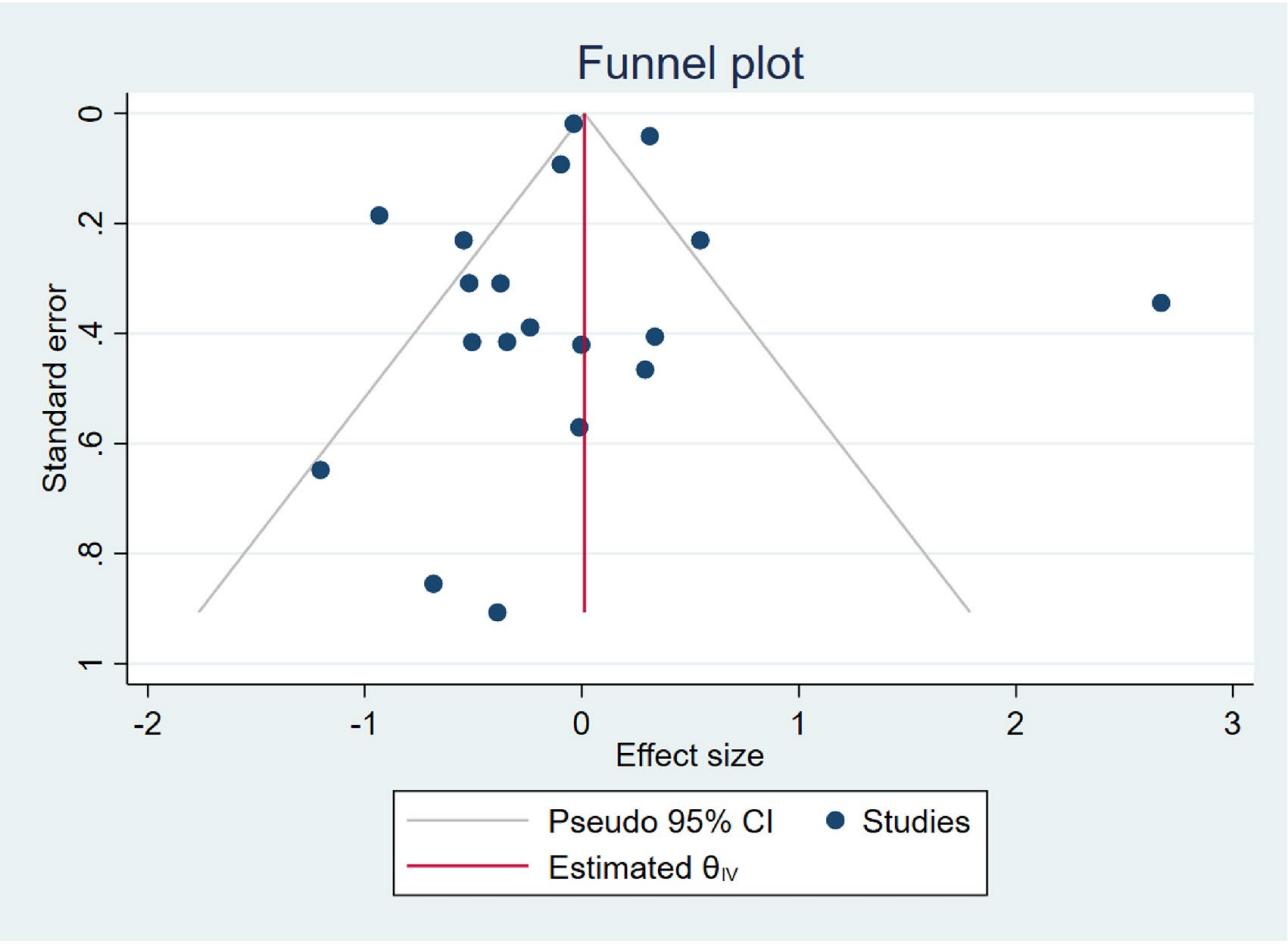
Funnel plot of the studies assessing GLP-1 use compared to other treatments.

Figure 4 shows the funnel plot of the included studies. Visual inspection of the funnel plot showed a symmetrical distribution of studies around the pooled effect size, suggesting no apparent publication bias. This was further supported by Egger’s regression test (*β* = −0.55, SE = 0.92, *p* = 0.55), indicating no significant small-study effects (Figure 5).

#### Risk of bias assessment

**Figure 6:**
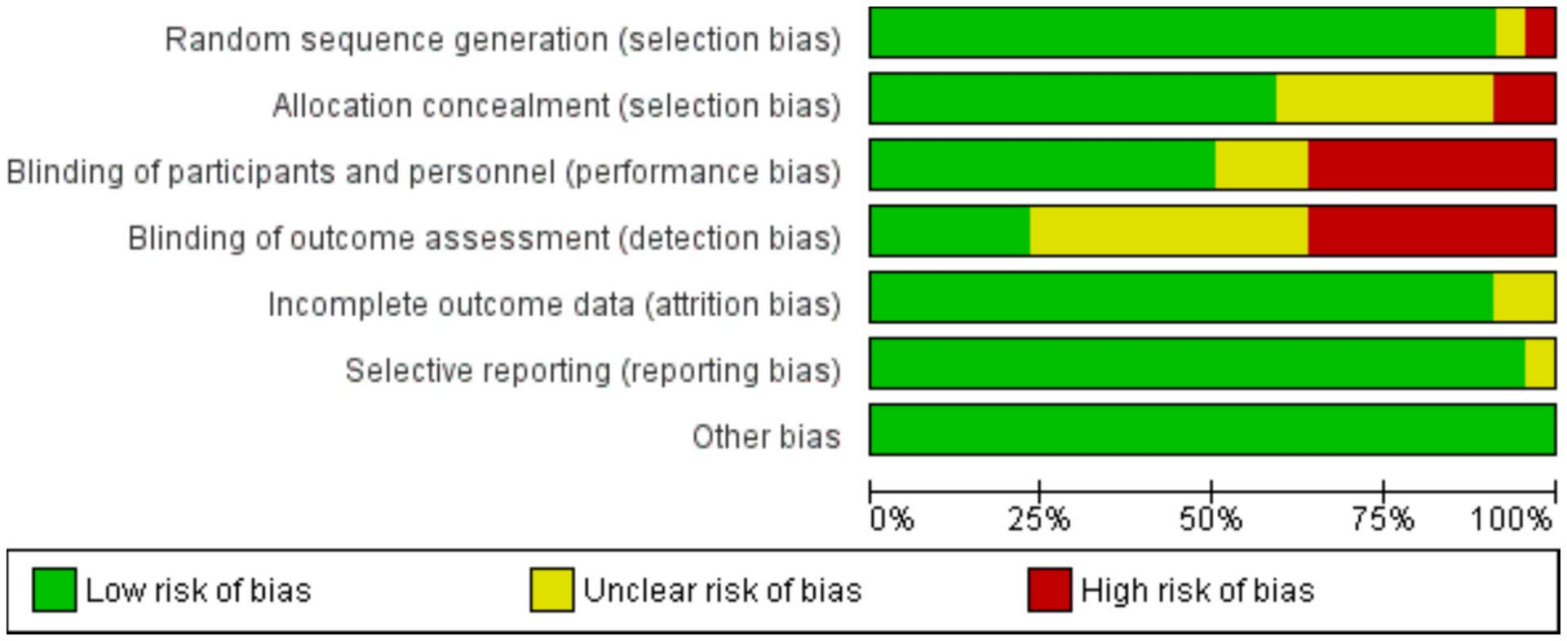
Risk of bias graph, presented as % across all included studies.

The Cochrane risk of bias was assessed for RCT studies (Table 2). In total 24 RCTs were included, 14 trials were considered to be at low risk of bias for all domains, and 10 trials were considered at high risk of bias (Figure 6). For the observational studies, the NOS quality assessment of 15 studies is summarized in (Table 1). 12 studies were given more than six stars and ranked as high-quality. On the other hand, 3 studies were given six to four stars and were considered of moderate quality. Regarding the quality of the studies included, one of the included studies did not address how treatment randomization occurred (31), how allocation of treatment was concealed was not mentioned in two studies (50, 51) and therefore had a high risk of selection bias, as shown in Figure 5 and Supplementary Figure S4. Eight studies did not describe the blinding strategy with sufficient details (26, 32, 38, 39, 47, 50, 53, 54) whereas the risk of reporting bias was minimum in all studies.

**Table 2:** Quality assessment for clinical trials.

The Cochrane risk-of-bias assessment tool for randomized trials								
Study ID	Random sequence generation (selection bias)	Allocation concealment (selection bias)	Blinding of participants and personnel (performance bias).	Blinding of outcome assessment (detection bias)	Incomplete outcome data (attrition bias)	Selective reporting (reporting bias)	Other bias	Overall bias
Vanita R. Aroda, 2019 (22)	Low risk	Low risk	Low risk	Low risk	Low risk	Low risk	Unclear risk	Unclear risk
M. S. Capehorn, 2019 (26)	Low risk	Unclear risk	High risk	High risk	Low risk	Low risk	Unclear risk	High risk
Melanie Davies, 2021 (29)	Low risk	Low risk	Low risk	Unclear risk	Low risk	Low risk	Unclear risk	Unclear risk
Ruth Cordiner, 2016 (30)	Low risk	Unclear risk	Low risk	Unclear risk	Unclear risk	Unclear risk	Unclear risk	Unclear risk
Ji, L., 2020 (31)	Low risk	Low risk	Low risk	Unclear risk	Low risk	Low risk	Unclear risk	Unclear risk
Nobuya Inagaki, 2011 (32)	Low risk	Unclear risk	High risk	High risk	Low risk	Low risk	Unclear risk	High risk
Adrian F. Hernandez, 2018 (33)	Low risk	Low risk	Low risk	Low risk	Low risk	Low risk	Unclear risk	Unclear risk
Ildiko Lingvay, 2019 (36)	Low risk	Low risk	Low risk	Low risk	Low risk	Low risk	Unclear risk	Unclear risk
Bernhard Ludvik, 2021 (37)	Low risk	Low risk	Unclear risk	Unclear risk	Low risk	Low risk	Unclear risk	Unclear risk
Kohei Kaku, 2016 (38)	Low risk	Low risk	High risk	High risk	Low risk	Low risk	Unclear risk	High risk
Kohei Kaku, 2011 (39)	Low risk	Unclear risk	Unclear risk	Unclear risk	Low risk	Low risk	Unclear risk	Unclear risk
Kaku, K, 2018 (40)	Low risk	Low risk	High risk	High risk	Low risk	Low risk	Unclear risk	High risk
Juan P. Frías, 2021 (42)	Low risk	Unclear risk	Low risk	Unclear risk	Low risk	Low risk	Unclear risk	Unclear risk
Marso, S. P., 2016 (13)	Low risk	Low risk	Low risk	Low risk	Low risk	Low risk	Unclear risk	Unclear risk
Bernard Zinman, 2019 (46)	Low risk	Low risk	Low risk	Low risk	Low risk	Low risk	Unclear risk	Unclear risk
Daisuke Yabe, 2020 (47)	Low risk	Low risk	High risk	High risk	Low risk	Low risk	Unclear risk	High risk
Watada, H., 2019 (48)	Low risk	Low risk	Low risk	Low risk	Low risk	Low risk	Unclear risk	Unclear risk
Seino, Y., 2018 (50)	Low risk	Low risk	High risk	High risk	Low risk	Low risk	Unclear risk	High risk
Yutaka Seino, 2016 (51)	Low risk	High risk	Low risk	Unclear risk	Low risk	Low risk	Unclear risk	High risk
Julio Rosenstock, 2019 (52)	Low risk	Low risk	Low risk	Low risk	Low risk	Low risk	Unclear risk	Unclear risk
Pratley, R. E., 2018 (53)	Low risk	Low risk	High risk	High risk	Low risk	Low risk	Unclear risk	High risk
Pieber, T. R., 2019 (54)	Low risk	Low risk	High risk	High risk	Low risk	Low risk	Unclear risk	High risk
Yukiko Onishi, 2013 (55)	Low risk	Unclear risk	High risk	High risk	Low risk	Low risk	Unclear risk	High risk
Inaha Okuda, 2017 (56)	High risk	Unclear risk	High risk	High risk	Low risk	Low risk	Unclear risk	High risk

## Discussion

GLP-1 receptor agonist medications as a class have become an important class of medications for managing type 2 diabetes and obesity, offering benefits such as improved glycemic control, weight loss, low risk of hypoglycemia and improved cardiovascular health. There were some concerns linking GLP-1RA with accelerated progression of DR raised by the SUSTAIN-6 secondary to the rapid improvement/reduction of blood glucose and glycemic control. Marso et al. (13), while other studies showed improved prognosis of DR in patients using GLP-1RA (17, 18). Our review attempted to investigate the relationship between DR outcomes with GLP-1 receptor agonists treatment by systematically examining and meta-analyzing the studies that looked at the impact of those medications on DR from inception until March 2025.

Our findings showed that GLP-1 receptor agonists were not significantly associated with the risk of DR compared with comparator groups (pooled RR = 1.00, 95% CI 0.71–1.43). In a study conducted by Jiao et al. (57) GLP-1 receptor agonists are not associated with an increased risk of DR. Our analysis suggested that semaglutide intervention was associated with a lower risk of developing DR compared with the control group, with a pooled risk ratio of 0.86, (95% CI 0.62–1.20) This result should be interpreted with caution, as the included studies had variable follow-up durations, which may have limited the precision of the estimate. In previous study conducted by Wang et al. (58) individuals aged over 60 years and with a diabetes duration of more than 10 years were identified as being at higher risk of developing DR when using semaglutide.

The association between the progression of diabetic retinopathy (DR) and GLP-1 receptor agonist (GLP-1RA) use is based on the presence of GLP-1 receptors on the retina. This association, however, remains contradictory. The trials conducted can be generally categorized into systemic and topical GLP-1 administration. Topical administration of GLP-1 liraglutide via eye drops on experimental mice models at 10 weeks old of early diabetic stage revealed various protective mechanisms on the retina including: (1) anti-apoptotic effects and anti-inflammatory actions via preventing the upregulation of pro-inflammatory factors, (2) preservation of the blood-retinal barrier and most importantly, (3) significant downregulation of extracellular diabetic glutamate (12, 59).

In another study conducted on similar mice models at 24 weeks old, it confirms the arrest of progression of retinal neurodegeneration and reversal of pathogenic mechanisms particularly through its anti-inflammatory and VEGF downregulation activity (60). In another recent study conducted by Oezer et al., using lixisenatide on DR, the results concurred with the aforementioned protective effects and further revealed antioxidative effects, mitigation of macro and microglial activation and normalization of diabetic gene expressions to control levels (61). Several other studies have been conducted on topical GLP-1 administration supporting the previously mentioned effects and providing other protective mechanisms including mediation of mitophagy, neuronal proliferation and supporting DNA repair. In light of these studies, GLP-1 agonist has been proposed as a potential DR therapeutic option due to its beneficial local effects independent of their impact on blood glucose control (60, 62–64).

Other clinical studies Several meta-analyses and observational cohorts align partly with our finding of a non-statistically significant association between GLP-1 receptor agonist (GLP-1RA) use and development or progression of diabetic retinopathy (DR). For instance, a recent systematic review and meta-analysis including RCTs found no significant difference in DR complication risk. When comparing GLP-1RAs to other treatments (57, 65). Another real-world observational study of sight-threatening DR in US adults initiating GLP-1RAs found no significant difference overall, but importantly these analyses emphasized that rapid glycemic improvement may contribute to early worsening of DR, particularly in patients with pre-existing retinopathy (66). In light of this, the exact relationship between GLP-1 and DR is not fully understood, Therefore, it is thought that future research is needed to confirm the effects and determine the optimal use of GLP-1 agonists in individuals with DR.

It’s essential for individuals with diabetes, includingthose at risk for retinopathy, to receive regular eye examinations and comprehensive diabetes management to help prevent or manage this complication in addition to the management of other risk factors for DR.

### Implication for practice

The study indicates that the incidence of diabetic retinopathy was comparable between the GLP-1 receptor agonist and control groups (pooled RR = 1.00, 95% CI 0.71–1.43). This suggests that GLP-1 receptor agonists do not significantly affect the overall risk of developing or progressing diabetic retinopathy. Physicians should continue to monitor patients with pre-existing retinopathy and maintain comprehensive glycemic control as the main strategy for preventing disease progression.

### Implication for policy

Since GLP-1 receptor agonists showed no significant effect on diabetic retinopathy risk, policies should focus on broader diabetes management strategies that strengthen prevention, early detection, and integrated care for diabetic complications. This approach ensures comprehensive care and emphasizes the importance of regular screening for DR, guiding policy development toward more effective and holistic diabetes care strategies.

### Implication for future research

The study underscores the importance of further research to conclusively determine the long-term effects of GLP-1RA on the incidence of DR. Future studies with larger sample sizes, longer follow-up duration, and diverse populations are needed to better understand the potential benefits of GLP-1RA in preventing DR in patients with DM.

However, there are a few limitations of our current work. The limitations of this study are as follows. First, most of the included studies did not assess ocular safety beyond 1 year; therefore, the findings should be interpreted with caution when considering long-term effects. Secondly, due to differences in designs, populations, and comparators, the included studies are heterogeneous, which may affect the consistency of the results. However, we conducted subgroup analysis to investigate the source of heterogeneity. In addition, adjusting for particular patient features or risk factors may be more difficult when aggregate data is used instead of individual patient data. Future patient-level meta-analyses could clarify how baseline DR, HbA1c changes, and concomitant treatments affect ocular safety outcomes.

Furthermore, possible confounding variables may not have been properly taken into consideration.

## Conclusion

In conclusion, while this meta-analysis suggests a potential preventive effect of GLP-1 receptoragonists in DR compared to the control group, the results were not statistically significant. Further studies with longer follow-up and larger sample sizes are needed to better understand the impact of GLP-1RA on DR outcomes.

## Data Availability

The raw data supporting the conclusions of this article will be made available by the authors, without undue reservation.
