# Correction: Association of glucagon-like peptide-1 (GLP-1) receptor agonists and diabetic retinopathy (DR) – a systematic review and meta-analysis

**DOI:** 10.3389/fmed.2026.1778742

**Published:** 2026-02-05

**Authors:** Hassan Alwafi, Sarah Saleh Al-Harbi, Ghada Ahmad Aladwani, Safaa M. Alsanosi, Tope Oyelade, Fahd Almalki, Husna Irfan Thalib, Abdallah Y. Naser, Manal Z. Alfahmi, Basil AlOtaibi, Samra Fuad, Mohammed Talha Mohammed Zubair, Abdulelah M. Aldhahir, Widya N. Insani, Abdullah A. Alqarni, Jaber S. Alqahtani, Deema S. Ashoor, Mohammad Saleh Dairi

**Affiliations:** 1 Department of Pharmacology and Toxicology, College of Medicine, Umm Al-Qura University, Makkah, Saudi Arabia; 2 Department of Pharmacy, Umm Al-Qura University, Makkah, Saudi Arabia; 3 Institute for Liver and Digestive Health, UCL Division of Medicine, University College London, London, United Kingdom; 4 Department of Medicine, College of Medicine, Umm Al-Qura University, Makkah, Saudi Arabia; 5 General Medicine Practice Program, Batterjee Medical College, Jeddah, Saudi Arabia; 6 Department of Applied Pharmaceutical Sciences and Clinical Pharmacy, Faculty of Pharmacy, Isra University, Amman, Jordan; 7 Executive Administration of Research and Innovation, King Abdallah Medical City, Makkah, Saudi Arabia; 8 Independent Researcher, Jeddah, Saudi Arabia; 9 Respiratory Therapy Program, Department of Nursing, College of Nursing and Health Sciences, Jazan University, Jazan, Saudi Arabia; 10 Department of Pharmacology and Clinical Pharmacy, Padjadjaran University, Jatinangor, Indonesia; 11 Department of Respiratory Therapy, Faculty of Medical Rehabilitation Sciences, King Abdulaziz University, Jeddah, Saudi Arabia; 12 Department of Respiratory Care, Prince Sultan Military College of Health Sciences, Dammam, Saudi Arabia; 13 Al-Noor Specialist Hospital, Ministry of Health, Makkah, Saudi Arabia

**Keywords:** GLP-1 agonist, diabetes, obesity, diabetic retinopathy, meta-analysis, systematic review

Author Safaa M. Alsanosi was erroneously assigned to affiliation BHF Glasgow Cardiovascular Research Centre, Institute of Cardiovascular and Medical Sciences, University of Glasgow, Glasgow, United Kingdom. This affiliation has now been removed for author Safaa M Alsanosi.

The original version of this article has been updated.

