# Supplementary material for: Association of glucagon-like peptide-1 (GLP-1) receptor agonists and diabetic retinopathy (DR) – a systematic review and meta-analysis
Source: Front Med (Lausanne). 2025 Dec 18;12:1639704. doi: 10.3389/fmed.2025.1639704 (PMC12756447; doi:10.3389/fmed.2025.1639704)
Supplement: Supplementary file 1 [file Data_Sheet_1.docx]

**Supplementary Data**

**
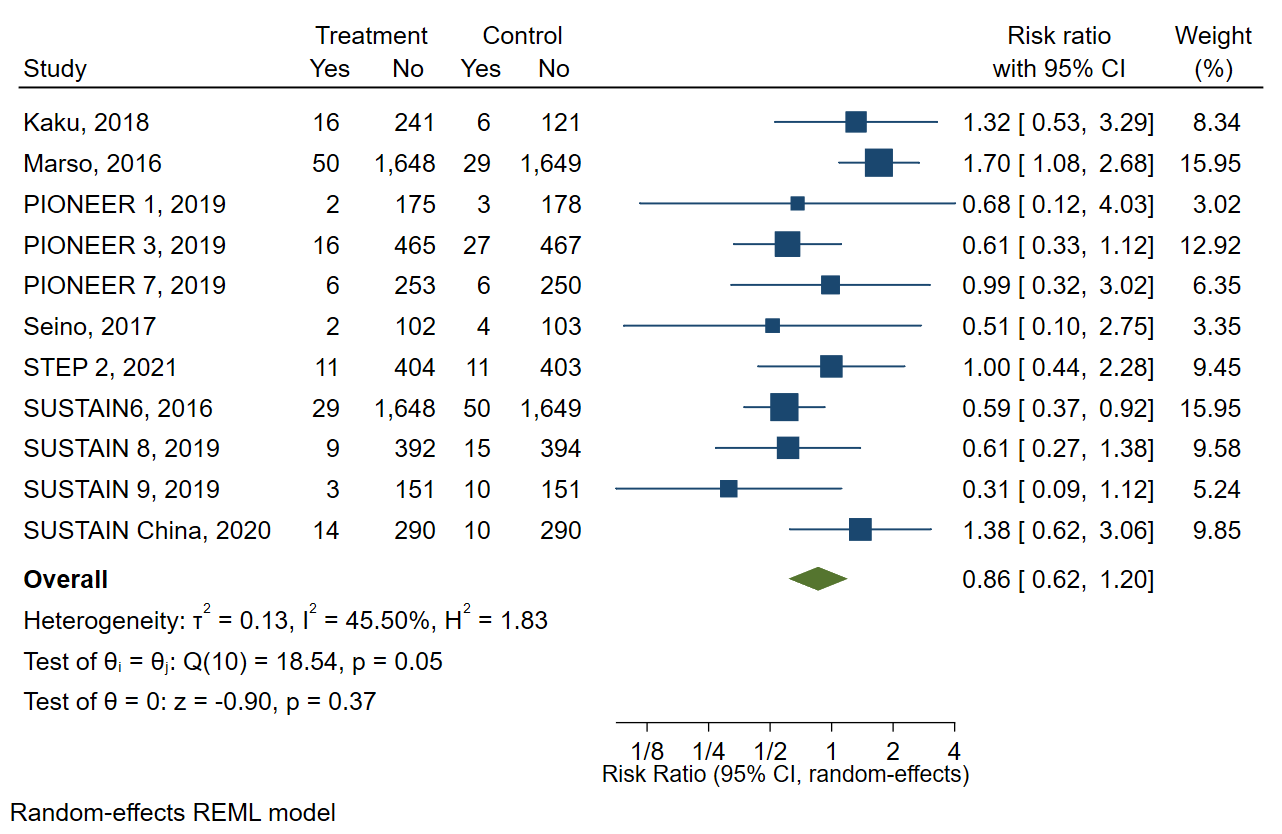
**

**Figure S1.** Subgroup analysis comparing semaglutide with other treatment group

In review

694
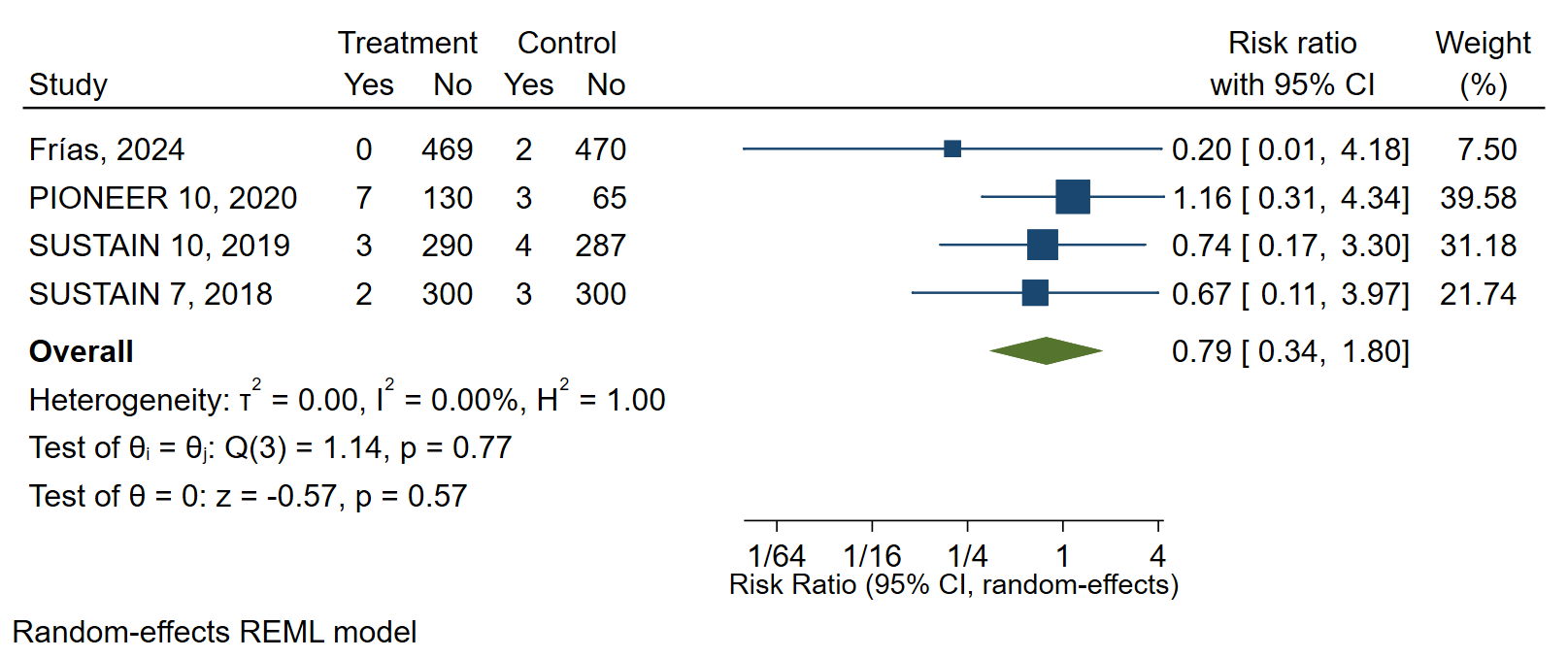


698 **Figure S2.** Subgroup analysis comparing semaglutide with other GLP-1 RA


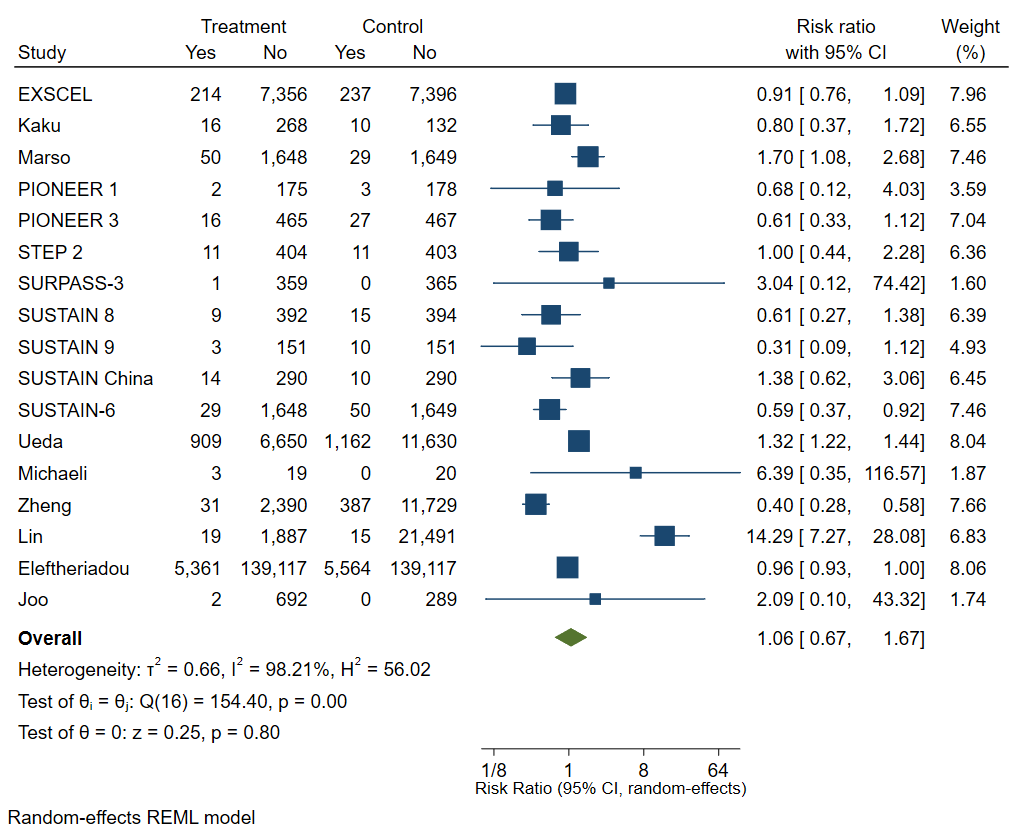


**Figure S3.** Sensitivity analysis by excluding studies with high risk of bias


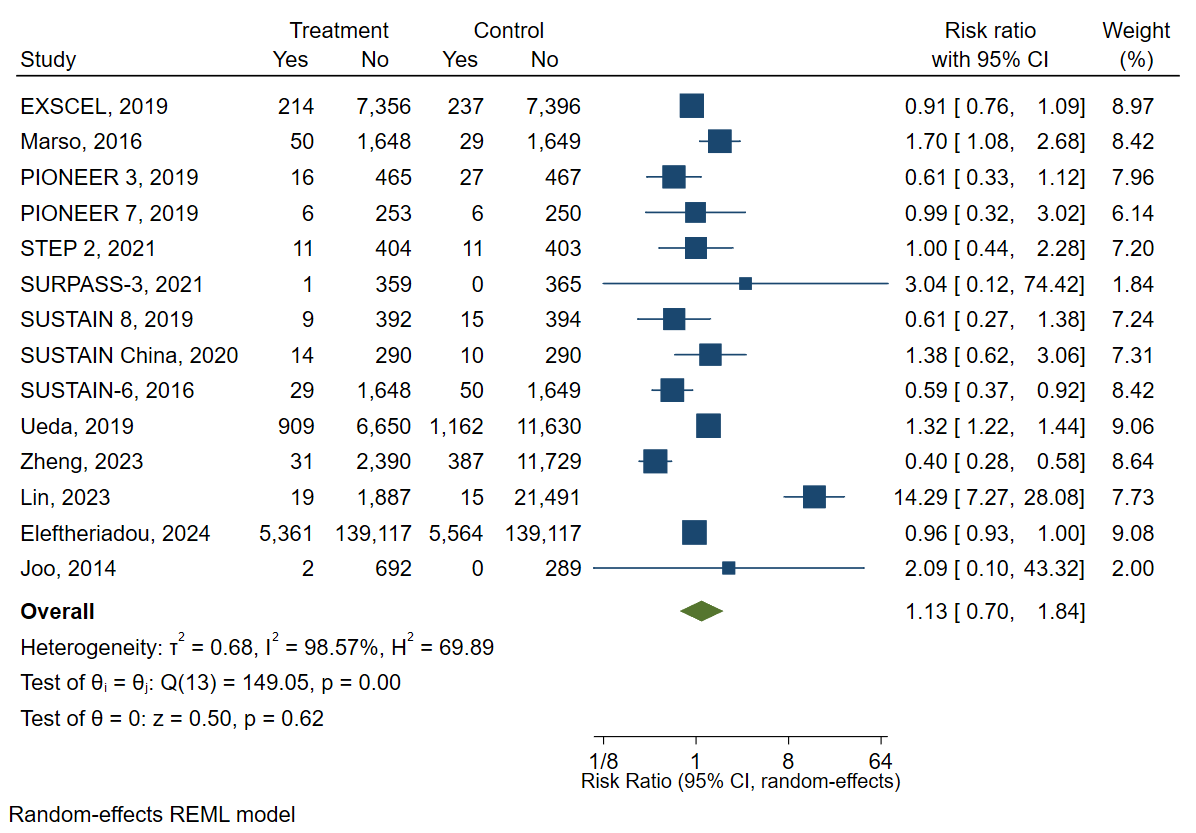


**Figure S4.** Sensitivity analysis restricted to studies with sample size ≥ 500

713


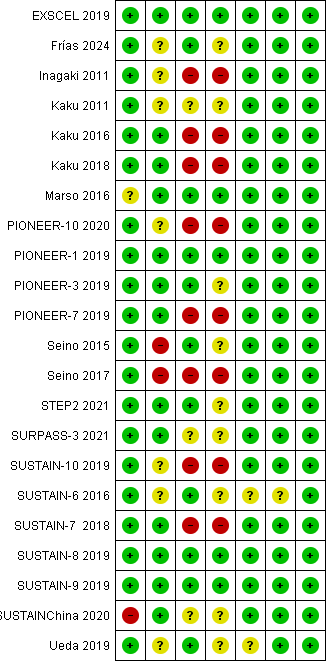
714

715

716

717

718

719

720

721

722

723

724

725

726

727

728

729

In review

730

731 **Figure S5**: Summary of risk of bias across randomized controlled trials

In review
