# Supplementary material for: Association of glucagon-like peptide-1 (GLP-1) receptor agonists and diabetic retinopathy (DR) – a systematic review and meta-analysis
Source: Front Med (Lausanne). 2025 Dec 18;12:1639704. doi: 10.3389/fmed.2025.1639704 (PMC12756447; doi:10.3389/fmed.2025.1639704)
Supplement: Supplementary file 2 [file Table_1.docx]

**Table S1**: Preferred Reporting Items for Systematic Reviews and Meta-Analyses (PRISMA) checklist.

| **Section and Topic** | **Item #** | **Checklist item** | **Location where item is reported** |
| --- | --- | --- | --- |
| **TITLE** | | |  |
| Title | 1 | Identify the report as a systematic review. | Page 1 |
| **ABSTRACT** | | |  |
| Abstract | 2 | See the PRISMA 2020 for Abstracts checklist. | Page 6 |
| **INTRODUCTION** | | |  |
| Rationale | 3 | Describe the rationale for the review in the context of existing knowledge. | Page 7,8 |
| Objectives | 4 | Provide an explicit statement of the objective(s) or question(s) the review addresses. | Page 7,8 |
| **METHODS** | | |  |
| Eligibility criteria | 5 | Specify the inclusion and exclusion criteria for the review and how studies were grouped for the syntheses. | Page 8-10 |
| Information sources | 6 | Specify all databases, registers, websites, organizations, reference lists and other sources searched or consulted to identify studies. Specify the date when each source was last searched or consulted. | Page 8-10 |
| Search strategy | 7 | Present the full search strategies for all databases, registers and websites, including any filters and limits used. | Page 8 |
| Selection process | 8 | Specify the methods used to decide whether a study met the inclusion criteria of the review, including how many reviewers screened each record and each report retrieved, whether they worked independently, and if applicable, details of automation tools used in the process. | Page 8 |
| Data collection process | 9 | Specify the methods used to collect data from reports, including how many reviewers collected data from each report, whether they worked independently, any processes for obtaining or confirming data from study investigators, and if applicable, details of automation tools used in the process. | Page 8-10 |
| Data items | 10a | List and define all outcomes for which data were sought. Specify whether all results that were compatible with each outcome domain in each study were sought (e.g. for all measures, time points, analyses), and if not, the methods used to decide which results to collect. | Page 8-10 |
|  | 10b | List and define all other variables for which data were sought (e.g. participant and intervention characteristics, funding sources). Describe any assumptions made about any missing or unclear information. | Page 4 |
| Study risk of bias assessment | 11 | Specify the methods used to assess risk of bias in the included studies, including details of the tool(s) used, how many reviewers assessed each study and whether they worked independently, and if applicable, details of automation tools used in the process. | Page 8-10 |
| Effect measures | 12 | Specify for each outcome the effect measure(s) (e.g. risk ratio, mean difference) used in the synthesis or presentation of results. | Page 8-10 |
| Synthesis methods | 13a | Describe the processes used to decide which studies were eligible for each synthesis (e.g. tabulating the study intervention characteristics and comparing against the planned groups for each synthesis (item #5)). | Page 8-10 |
|  | 13b | Describe any methods required to prepare the data for presentation or synthesis, such as handling of missing summary statistics, or data conversions. | Page 8-10 |
|  | 13c | Describe any methods used to tabulate or visually display results of individual studies and syntheses. | Page 8-10 |
|  | 13d | Describe any methods used to synthesize results and provide a rationale for the choice(s). If meta-analysis was performed, describe the model(s), method(s) to identify the presence and extent of statistical heterogeneity, and software package(s) used. | Page 8-10 |
|  | 13e | Describe any methods used to explore possible causes of heterogeneity among study results (e.g. subgroup analysis, meta-regression). | Page 8-10 |
|  | 13f | Describe any sensitivity analyses conducted to assess robustness of the synthesized results. | Page 8-10 |
| Reporting bias assessment | 14 | Describe any methods used to assess risk of bias due to missing results in a synthesis (arising from reporting biases). | Page 8-10 |
| Certainty assessment | 15 | Describe any methods used to assess certainty (or confidence) in the body of evidence for an outcome. | Page 8-10 |
| **RESULTS** | | |  |
| Study selection | 16a | Describe the results of the search and selection process, from the number of records identified in the search to the number of studies included in the review, ideally using a flow diagram. | Page 10,11 |
|  | 16b | Cite studies that might appear to meet the inclusion criteria, but which were excluded, and explain why they were excluded. | Page 10,11 |
| Study characteristics | 17 | Cite each included study and present its characteristics. | Page 10,11 |
| Risk of bias in studies | 18 | Present assessments of risk of bias for each included study. | Page 10,11 |
| Results of individual studies | 19 | For all outcomes, present, for each study: (a) summary statistics for each group (where appropriate) and (b) an effect estimates and its precision (e.g. confidence/credible interval), ideally using structured tables or plots. | Page 10,11 |
| Results of syntheses | 20a | For each synthesis, briefly summarize the characteristics and risk of bias among contributing studies. | Page 10,11 |
|  | 20b | Present results of all statistical syntheses conducted. If meta-analysis was done, present for each the summary estimate and its precision (e.g. confidence/credible interval) and measures of statistical heterogeneity. If comparing groups, describe the direction of the effect. | Page 10,11 |
|  | 20c | Present results of all investigations of possible causes of heterogeneity among study results. | Page 10,11 |
|  | 20d | Present results of all sensitivity analyses conducted to assess the robustness of the synthesized results. | Page 10,11 |
| Reporting biases | 21 | Present assessments of risk of bias due to missing results (arising from reporting biases) for each synthesis assessed. | Page 10,11 |
| Certainty of evidence | 22 | Present assessments of certainty (or confidence) in the body of evidence for each outcome assessed. | Page 10,11 |
| **DISCUSSION** | | |  |
| Discussion | 23a | Provide a general interpretation of the results in the context of other evidence. | Page 13 |
|  | 23b | Discuss any limitations of the evidence included in the review. | Page 14 |
|  | 23c | Discuss any limitations of the review processes used. | Page 14 |
|  | 23d | Discuss implications of the results for practice, policy, and future research. | Page 13,14 |
| **OTHER INFORMATION** | | |  |
| Registration and protocol | 24a | Provide registration information for the review, including register name and registration number, or state that the review was not registered. | Page 15 |
|  | 24b | Indicate where the review protocol can be accessed, or state that a protocol was not prepared. | Page 15 |
|  | 24c | Describe and explain any amendments to information provided at registration or in the protocol. | Page 15 |
| Support | 25 | Describe sources of financial or non-financial support for the review, and the role of the funders or sponsors in the review. | Page 15 |
| Competing interests | 26 | Declare any competing interests of review authors. | Page 15 |
| Availability of data, code and other materials | 27 | Report which of the following are publicly available and where they can be found template data collection forms; data extracted from included studies; data used for all analyses; analytic code; any other materials used in the review. | Page 15 |

*From:*  Page MJ, McKenzie JE, Bossuyt PM, Boutron I, Hoffmann TC, Mulrow CD, et al. The PRISMA 2020 statement: an updated guideline for reporting systematic reviews. BMJ 2021;372: n71. doi: 10.1136/bmj. n71. This work is licensed under CC BY 4.0. To view a copy of this license, visit <https://creativecommons.org/licenses/by/4.0/>

734 **Table S2**: Characteristics of Included Studies

| Characteristics of Included Studies | | | | | | | | | | |
| --- | --- | --- | --- | --- | --- | --- | --- | --- | --- | --- |
| Author | Year | Study  desig n | No. of  Participants | Age mean (SD) | Gender (male %) | Duration | Site of study | Ethnicity  (Hispanic or latino%) | Interv ention | Compa rison |
| Nielsen et al.  2022 [21] | 2022 | PCS | 48 | 49 (12) | 79% | 26  Weeks | NR | NR | liraglu  tide | placeb  o |
| Aroda VR et al. 2019 [22] | 2019 | RCT | 703 | 55 | 50.8% | 26-week | Algeria, Bulgaria, Czech Re- public, Japan, Mexico, Russia, Serbia,Tur key, and  the U.S. | 25.60% | Oral semag lutide (3,7,1  4 mg) | placeb o |
| Best JH et al. 2011 [23] | 2011 | RCS | 383,525 | Exenatide  = 52.7(8.7),  non- exenatide  =  53.2(11.2) | Exenatide  = 43.8%,  non- exenatide  = 51.5% | 9 months | U.S | NR | Exena tide b.i.d. | Non- exenati de |
| Bethel MA et al. 2020 [24] | 2020 | RCS | 14,752 | NR | NR | 3.2-  years | NR | NR | Once- Weekl y Exena  tide | placeb o |
| Blaslov K et al.  2013 [25] | 2013 | PCS | 91 | 58 (31-76) | 50.5% | 14  months | Zagreb,  Croatia. | NR | Exena  tide | NA |
| Capehorn MS et  al. 2020 [26] | 2019 | RCT | 576 | 59.5 | 56.7% | 30-week | Europe | NR | semag  lutide | liraglut  ide |

| Dauner DG et al. 2021 [27] | 2021 | RCS | 186,878 | NR | NR | NR | US Food and Drug Administr ation Adverse Event Reporting System | NR | (Sema glutid e, Dulag lutide, Exena tide, Liragl utide, Lixise natide  ,  Albigl utide). | NA |
| --- | --- | --- | --- | --- | --- | --- | --- | --- | --- | --- |
| Davidson JA et al. 2017 [28] | 2017 | RCS | 1204 | 52.2(10.3) | 42.4% | 26  weeks to 3 years | NR | 100% | albigl utide (30–  50  mg) | placeb o |
| Davies M et al. 2021 [29] | 2021 | RCT | 1210 | 55 (11) | 50.1% | 75  weeks | Europe, North America, South America, the Middle East, South Africa,  and Asi | 12·8% | once a week semag lutide 2·4 mg, semag lutide 1·0  mg, | placeb o |
| Douros A et al. 2018 [30] | 2018 | RCS | 77,115 | 61.6(13.6) | 57.3% | >12  months, | UK  Clinical Practice Research Datalink | NR | GLP- 1RAs  (exen atide, liraglu tide,  lixise | other an- tidiabet ic drugs / insulin |

In review

|  |  |  |  |  |  |  |  |  | natide  ) |  |
| --- | --- | --- | --- | --- | --- | --- | --- | --- | --- | --- |
| Cordiner et al. 2016 [31] | 2016 | RCT | 3297 | NR | NR | 109  weeks | NR | NR | once- weekl y semag lu- tide 0.5mg or  1.0mg | placeb o |
| Ji L et al. 2021 [32] | 2020 | RCT | 868 | 53 (11.4) | 55.6% | 30-week | Brazil, China, Republic of Korea, South Africa and  Ukraine | 9.40% | semag lutide 0.5  and 1.0  mg | sitaglip tin |
| Inagaki N et al 2011[33]. | 2011 | RCT | 152 | 59 (10) | 68% | 24-week | Japan | NR | Exena tide 5,10  ng,  b.i.d. | NR |
| Hernandez AF et al. 2018 [34] | 2018 | RCT | 9,463 | 64.1 (8.7) | 70% | 3·8 years. | North and South America, Europe, Africa,  and Asia. | NR | Albigl utide | placeb o |
| Guo L et al. 2022 [35] | 2022 | PCS | 3,294 | 50.1(13.2) | 67.9% | 24-week | China | NR | Dulag lutide | Dulagl  utide- na ı̈ve |
| Lin Y et al. 2023 [36] | 2023 | RCS | 8922 | 68.4(11.5) | 50.6% | 2.1 years | Taiwan | NR | GLP- 1RAs  includ  ed | DPP-4 |

In review

|  |  |  |  |  |  |  |  |  | liraglu tide and dulagl  utide |  |
| --- | --- | --- | --- | --- | --- | --- | --- | --- | --- | --- |
| Lingvay I et al. 2019 [37] | 2019 | RCT | 788 | 56.6(10.9) | 54% | 52-week | Argentina, Brazil, Canada, India, Ireland, Lebanon, Malaysia, Mexico, Sweden, the UK,  and the  USA | 37% | Sema glutid e 1·0 mg | Canagl iflozin 300 mg |
| Ludvik B et al. 2021 [38] | 2021 | RCT | 1444 | 57·4 (10·0) | 56% | 52  weeks | Argentina, Austria, Greece, Hungary, Italy, Poland, Puerto Rico, Romania, South Korea, Spain, Taiwan, Ukraine, and the  USA. | 30% | Tirzep atide 5, 10,  15 mg | Insulin deglud ec |
| Kaku K et al 2015 [39] | 2015 | RCT | 360 | 59.5(11.1) | 72.8% | 53  weeks | Japan | NR | Liragl utide | Liraglu tide +  Additi |

In review

|  |  |  |  |  |  |  |  |  |  | onal  OAD |
| --- | --- | --- | --- | --- | --- | --- | --- | --- | --- | --- |
| Kaku K et al 2011 [40] | 2011 | RCT | 400 | 58.3(10.4) | 67.3% | 52  weeks | Japan | NR | Liragl utide 0.9  mg/da  y | glibenc lamide 1.25–  2.5  mg/day |
| Kaku K et al 2018 [41] | 2018 | RCT | 601 | 58.5(10.3) | 71.5% | 56  weeks. | Japan | NR | Sema glutid e 0.5 ,  1.0  mg | Semagl utide + Additi onal  OAD |
| Fadini GP et al. 2018 [42] | 2018 | RCS | 9,217,555 | NR | NR | NR | NR | NR | ‘insuli n deglu dec and liraglu tide’ or ‘liragl utide’ OR  ‘exen atide’ OR  ‘lixise natide ’ OR  ‘albigl utide’ OR  ‘dulag lutide’ OR  ‘tedug | insulin s and other medica tions |

In review

|  |  |  |  |  |  |  |  |  | lutide’  . |  |
| --- | --- | --- | --- | --- | --- | --- | --- | --- | --- | --- |
| Frías JP et al. 2021 [43] | 2021 | RCT | 1878 | 56.6(10.4) | 47% | 40-week | United States, Argentina, Australia, Brazil, Canada, Israel, Mexico, and the United  Kingdom | 70.10% | Tirzep atide 5, 10 ,  15 | Semagl utide |
| Lin TY et al, 2022 [44] | 2022 | RCS | 23,378 | 61.0(10.3) | 59.7% | 1.74  ±1.13  years | Chang Gung Research Database, Taiwan | NR | GLP1  -RAs  (dulag lutide and liraglu  tide) | SGLT2 |
| Marso SP et al. 2016 [45] | 20  16 | RCT | 3297 | 64.6 (7.4) | 60.7% | 104  weeks | NR | NR | once- weekl y semag lutide (0.5  mg or 1.0  mg) | placeb o |
| Ueda P et al 2019 [46] | 2019 | RCS | 18,280 | NR | NR | 6 months | Denmark , Sweden | NR | liraglu tide, exenat ide, dulagl utide,  and | DPP4i |

In review

|  |  |  |  |  |  |  |  |  | lixise  natide |  |
| --- | --- | --- | --- | --- | --- | --- | --- | --- | --- | --- |
| Wang T et al 2018 [47] | 2018 | RCS | 92,444 | 73.0(5.17) | 42.3% | NR | U.S. | NR | GLP- 1 RA | long acting  insulin |
| Zheng D et al. 2023 [48] | 2023 | RCS | 14,119 | GLP-1 RA: 52.65  (10.38) ,  non-GLP-  1 RA:  53.30  (10.37) | 55.20% | NR | Sweden | NR | GLP- RA | NON- GLP- 1RA |
| Zinman B et al. 2019 [49] | 2019 | RCT | 302 | 57.0 (9.5) | 58.3% | 30-week | Austria, Canada, Japan, Norway, Russia, and the  USA | 7·3% | Sema glutid e 1·0 mg | placeb o |
| Yabe D et al. 2020 [50] | 2020 | RCT | 458 | 58 (10) | 74% | 57  weeks | Japan | NR | Oral semag lutide 3,7,14  mg | Dulagl utide 0·75  mg |
| Watada H et al. 2019 [51] | 2019 | RCT | 210 | 56.0(10.2) | 63.3% | 26  weeks | Japan | NR | insuli n deglu dec and liraglu tide (IDeg  Lira) | insulin deglud ec |
| Sullivan SD et al 2009 [52] | 2009 | RCS | 5000 | 56.1 (9.8) | NR | 30 years | NR | NR | Liragl  utide  1.8 , | Rosigli  tazone 4mg |

In review

|  |  |  |  |  |  |  |  |  | 1.2  mg |  |
| --- | --- | --- | --- | --- | --- | --- | --- | --- | --- | --- |
| Seino Y et al 2017 [53] | 2017 | RCT | 308 | 58.3(10.7) | 76.3% | 30  weeks | Japan | NR | once- weekl y subcut ane (s.c.) semag lutide (0.5 or  1.0  mg) | once- daily oral sitaglip tin 100 mg. |
| Seino Y et al. 2016 [54] | 2016 | RCT | 257 | 60.5(11.2) | 56% | 36  weeks | Japan | NR | Liragl utide 0.9  mg/da y +  insuli  n | Liraglu tide Placeb o +  insulin |
| Rosenstock J et al. 2019 [55] | 2019 | RCT | 1864 | 58 (10.0) | 52.7% | 78  weeks | Japanese or non- Japanese | 16.60% | Oral Sema glutid e 3,7,14  mg/d | Sitagli ptin, 100  mg/d |
| Pratley RE et al. 2018 [56] | 2018 | RCT | 1201 | 55 (10·6) | 56% | 40-week | Bulgaria, Croatia, Finland, Germany, Greece, Hong Kong, India, Ireland,  Latvia, | 12% | Sema glutid e 0·5  or 1 mg | Dulagl utide 0·75 or  1.5 mg |

In review

|  |  |  |  |  |  |  | Lithuania, Portugal, Romania, Slovakia, Spain, the UK, and  the USA. |  |  |  |
| --- | --- | --- | --- | --- | --- | --- | --- | --- | --- | --- |
| Pieber TR et al. 2019 [57] | 2019 | RCT | 504 | 56.9 (9.7) | 57% | 52-week | Argentina, Austria, Belgium, Brazil, Egypt, Norway, South Korea, Switzerlan d, Turkey, USA | 21% | semag lutide with flexibl e dose adjust ments to 3,  7, or  14 mg once daily | sitaglip tin 100 mg once daily. |
| Onishi Y et al. 2013 [58] | 2013 | PCS | 155 | 61 (10) | 61% | 52-week | Japan | NR | exenat ide twice daily switch ed to exenat ide once weekl  y | exenati de once weekly |
| Okuda I et al. 2017 [59] | 2017 | PCS | 374 | 57.7 (9.9) | 71.1% | 1-year | Japan | NR | albigl utide 30 mg once- weekl  y | NR |

In review

In review

| 735 | RCT: randomized control trial, RCS: retrospective cohort study, PCS: prospective cohort study, |
| --- | --- |
| 736 | NR: Not Reported |
